# Empirical estimation of habitat suitability for rare plant restoration in an era of ongoing climatic shifts

**DOI:** 10.1038/s41598-023-46793-7

**Published:** 2023-11-07

**Authors:** Paul D. Krushelnycky, Lucas Berio Fortini, Jeffrey Mallinson, Jesse M. Felts

**Affiliations:** 1 Department of Plant and Environmental Protection Sciences, University of Hawaiʻi at Mānoa, Honolulu, HI USA; 2 U.S. Geological Survey, Pacific Island Ecosystems Research Center, Honolulu, HI USA; 3 Resources Management Division, Haleakalā National Park, Makawao, HI USA

**Keywords:** Ecology, Plant sciences, Climate sciences

## Abstract

Accurate estimates of current and future habitat suitability are needed for species that may require assistance in tracking a shifting climate. Standard species distribution models (SDMs) based on occurrence data are the most common approach for evaluating climatic suitability, but these may suffer from inaccuracies stemming from disequilibrium dynamics and/or an inability to identify suitable climate regions that have no analogues within the current range. An alternative approach is to test performance with experimental introductions, and model suitability from the empirical results. We used this method with the Haleakalā silversword (*Argyroxiphium sandwicense* subsp. *macrocephalum*), using a network of out-plant plots across the top of Haleakalā volcano, Hawaiʻi. Over a ~ 5-year period, survival varied strongly across this network and was effectively explained by a simple model including mean rainfall and air temperature. We then applied this model to estimate current climatic suitability for restoration or translocation activities, to define trends in suitability over the past three decades, and to project future suitability through 2051. This empirical approach indicated that much of the current range has low suitability for long-term successful restoration, but also identified areas of high climatic suitability in a region where plants do not currently occur. These patterns contrast strongly with projections obtained with a standard SDM, which predicted continued suitability throughout the current range. Under continued climatic shifts, these results caution against the common SDM presumption of equilibrium between species’ distributions and their environment, even for long-established native species.

## Introduction

Numerous species are predicted to have difficulty adapting quickly enough or dispersing far enough to track climate change-induced shifts in habitat suitability^1–4^. Translocation may thus be needed to avoid population or species decline and even extinction^5,6^. Such “assisted migration” is not without some controversy^7^, but the risk of unintended consequences from assisted species movement is in part dependent on the scale of movement. Translocations to nearby regions are expected to involve lower risk than introductions to more distant and presumably distinct ecological communities, and restoration within a species’ former range is expected to pose the least risk to other vulnerable species^5,8,9^. Accurate estimation of locations of current and future climatic suitability can therefore help identify the full range of management options for species facing climate impacts.

Most climatic suitability projections are based on correlative species distribution models (SDMs) that estimate a species’ climate envelope from occurrence data^10–12^. However, current distributions may not accurately reflect climatic suitability for several reasons. If populations of a species are currently declining from climate stress but have not yet disappeared from those regions, the suitable climate envelope may be overestimated. On the other hand, if a species’ distribution has contracted historically for non-climatic reasons, or is otherwise limited by dispersal^13^, the suitable climate envelope may be underestimated. Furthermore, a central assumption of niche uniformity made by standard SDMs is violated by the many species that exhibit local adaptation^14,15^. To overcome some of these limitations and better estimate current and future potential ranges, a variety of modified SDMs attempt to incorporate physiological or demographic processes and may also include intra-specific variation in environmental tolerances^16–19^.

As an alternative, experimental introductions test for survival or other demographic performance metrics across climatic gradients, potentially exceeding the current range^20,21^, and the resulting data provide a rigorous basis from which to model suitability. This is an extension of methods, such as reciprocal out-planting experiments or provenance tests, that assess performance and potential local adaptation across a species’ distribution^22–25^. For species that can easily be tracked and removed if needed, such as plants and other largely sessile organisms, the analysis of demographic performance in experimental introductions may offer a powerful method for predicting climatic suitability across a range of candidate habitats. It may, for example, accurately identify areas of suitable habitat that have no climatic analogues within the current distribution (i.e., reveal a wider environmental niche for the species), an advantage over modelling methods that have questionable performance when extrapolating in environmental space^26^. When species declines are thought to result from increasing climatic mismatch, the assessment of habitat suitability beyond the current range can become especially important.

We used this empirical approach to estimate climatic suitability for the Haleakalā silversword, or ʻāhinahina (*Argyroxiphium sandwicense* subsp. *macrocephalum*), a Hawaiian alpine giant rosette plant on Haleakalā volcano, Maui, that has been undergoing substantial climate-associated decline over the past several decades^27–29^. The entire population is estimated to have declined in abundance by approximately 60% since around 1990, but has not yet appreciably contracted in distribution despite most of this mortality occurring in the lower half of its range^28^. At the same time, the historical range of the Haleakalā silversword is known to be considerably larger than its current distribution, having contracted in the late 19th to early twentieth centuries as a result of over-harvesting and grazing by domesticated and feral ungulates^30^. The Haleakalā silversword thus represents a good example of how the current distribution, and even the known recorded distribution, may not accurately represent current climatic suitability.

Rainfall and air temperature have consistently been found to be strongly associated with recent silversword demographic patterns and trends, as well as with individual plant physiology and survival^27–29,31^. Although its population trajectory resulting from changes in these and related climate parameters is concerning, the species is readily amenable to restoration through out-planting. The goal of this study, therefore, was to evaluate relative climatic suitability of habitat for out-planting of silverswords across the top of Haleakalā volcano, including areas outside the current range. Because of the strong past linkages of silversword dynamics with rainfall and air temperature, we established a network of sites that encompassed the majority of the gradients in these two climate variables within the target region. At each site, we out-planted silverswords, collected local climate data, and tracked out-plants over nearly 5 years to evaluate climatic associations with survival and growth. These associations were then used to model spatial patterns and temporal trends in climatic suitability to aid present and future conservation actions. Finally, we compared these results to those obtained from a standard SDM based on recent occurrence data, to illustrate important differences between the two approaches.

## Materials and methods

### Study species and study design

**Figure 1:**
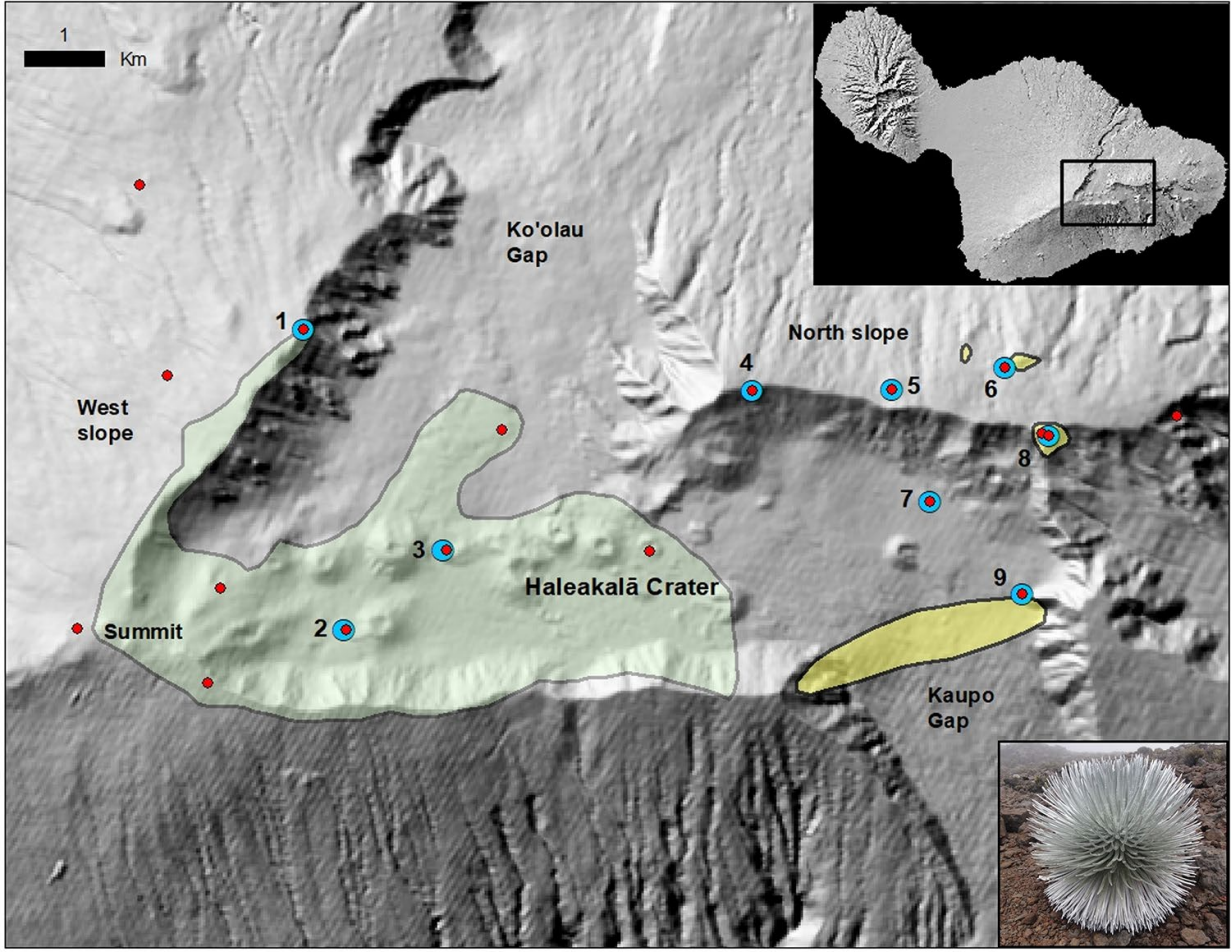
Map of study area, showing current silversword range (light green polygon), additional areas occupied historically (yellow polygons), the nine out-plant sites (blue circles), and 16 climate stations in the region (red dots). Note that the large yellow polygon represents a rough estimate based on a very brief and imprecise description [33]. The three smaller yellow polygons on the north slope are based on more recent and precise information. Plants reportedly also grew in unspecified locations on the upper west slope of the volcano in the nineteenth century. Inset shows region of Maui figured. Numbered out-plant sites are: 1 = Leleiwi, 2 = Puʻu o Pele, 3 = Lava Lake, 4 = Hanakauhi, 5 = Aloha Park, 6 = Puʻu Alaea, 7 = Laie, 8 = Pohaku Palaha, 9 = Paliku. Image of silversword at bottom right. Map created using ArcGIS software version 10.8.2, https://www.esri.com/en-us/arcgis/products/arcgis-desktop/overview.

The Haleakalā silversword is a federally listed threatened taxon in the family Asteraceae that occurs only on East Maui, Hawaiʻi. The Haleakalā silversword is a long-lived (mean lifespan > 20 years^29^), monocarpic (i.e., dies after flowering), self-incompatible rosette plant. Its distribution today includes the largely barren cinder cones, cinder flats, and rocky cliffs in a broad geographic area (roughly 1900 ha) from the central portions of Haleakalā Crater west up to the summit, as well as portions of the outer western and southern crater rims (Fig. 1). This current range spans elevations from 2155 to 3050 m, representing estimated long-term mean annual air temperatures (MAT) of 7.1 to 9.7 °C, and long-term mean annual precipitation (MAP) of 1015 to 1350 mm (long-term means calculated from 1978 to 2007^32^). Reported past silversword localities include areas on the northern and eastern rims of the crater, the north slope of the volcano, and on steep slopes in upper Kaupo Gap in the southeastern portion of the crater (Fig. 1^33–35^). These areas are located in more windward, wetter locations, resulting in a historical distribution that encompasses a larger climate range, which based on the same long-term averages spans approximately 7.1 to 10.5 °C MAT and 1015 to 3575 mm MAP. Silverswords were also reported to grow on the upper western slope in the nineteenth century^33,36,37^, and while exact locations were unspecified, this region likely falls within the larger historical climate range given above.

To evaluate the widest possible range of climatic conditions for potential silversword restoration, we selected nine sites that (1) spanned almost the entirety of the available gradients in estimated long-term MAT and MAP that occur above tree line or thick grassland on the top of Haleakalā volcano, and (2) possessed the lowest possible correlation between these two climate variables, allowing us to effectively separate the role of each variable and potentially other related climate variables. We further limited the sites to patches of bare ground because the vast majority of plants currently grow in largely unvegetated habitats, and we wanted to avoid confounding dynamics related to plant competition or facilitation in this study. The resulting nine sites (Leleiwi, Puʻu o Pele, Lava Lake, Hanakauhi, Aloha Park, Puʻu Alaea, Pohaku Palaha, Paliku, and Laie) are shown on Fig. 1 (refer to Fig. S1 and additional information on site selection in Supplementary Information).

At each site, we deployed a small climate station prior to out-planting, and collected air temperature (AT), rainfall (RF), relative humidity (RH) and soil moisture (SM) data every 10 min using Onset Computer Corporation components: H21-002 datalogger with S-RGB-M002 rain gauge, S-THB-M002 AT/RH sensor, and S-SMD-M005 Decagon Devices 10HS 160 × 32 mm SM sensor. Ten-minute data points were averaged (or summed in the case of RF) into hourly data, and hourly vapor pressure deficit (VPD) was calculated from hourly AT and RH data according to Lowe^38^. Daily means of AT, RH, SM, and VPD and daily sums of RF were then calculated from the hourly data for each day during the study period that had at least 23 h of data. Daily means for missing days were estimated for each station using gap-filling procedures, using data from the other sites, from four additional stations that contribute to the CraterNet network, and four stations from the HaleNet network (Fig. 1^39^). For each climate variable, missing data were filled using linear regression relationships with the station with the highest correlation coefficient for that variable. For RF, a no-rain condition was also imposed, in which zero rainfall was estimated when the daily total at the best-correlated station was zero^40^.

### Plant propagation, out-planting and monitoring

Seeds were collected from five maternal plants in each of two areas located at mid and high elevations in the silversword range in September of 2015: Keʻoneheʻeheʻe Trail at about 2480 m elevation, and the south rim of the crater at about 2920 m elevation. Seeds were refrigerated until they were sowed in flats containing potting soil mix (1:1:1 fine cinder:perlite:vermiculite) and approximately 10 g of slow release fertilizer pellets (Nutricote Total 13-13-13, Type 180, Chisso-Asahi Fertilizer Co.) on 5 February 2016, and were watered twice daily for 15 min by overhead sprinklers in a large greenhouse located at 2070 m elevation. Seedlings were transferred to pots (approx. 10 × 10 × 10 cm) containing the same soil medium and approximately 0.9 g of slow release fertilizer on 8 and 9 May 2016. Owing to differences in germination rates among maternal plants, different numbers of sibling seedlings were available for out-planting. However, seedling assignment to each of the nine out-plant plots was balanced with respect to maternal plant, with each plot receiving between two and eight siblings from each maternal plant. Forty-two plants were allocated to each plot (378 total), 14 of which derived from mid elevation maternal plants and 28 from high elevation maternal plants. The watering regime for the potted plants was changed to once daily for 15 min on 31 May 2016, once every Monday, Wednesday and Friday for 15 min on 9 September 2016, and once every Monday and Thursday for 15 min on 24 October 2016.

All out-planting was completed on 23 to 25 January 2017. Plants were situated approximately 1 m apart in randomized order along short transects within each plot and received 750 ml of water immediately after planting to reduce the effects of transplant shock^41^, with no further watering. Each site was subsequently monitored at the end of each wet season (November–April) and dry season (May–October) through the end of the fifth dry season, or 57 months, post-planting (November of 2021), except for the end of the fourth wet season in May of 2020, which was missed. During each monitoring event, we measured maximum rosette diameter of live tissue to the nearest centimeter and recorded survival status. We calculated relative growth rate (RGR) from the time of planting as (ln(final diam^2^)-ln(initial diam^2^))/(growth period in days). No live plant collection was done; identification of seed source material was made by Paul Krushelnycky and Jeffrey Mallinson; seed collection, plant propagation, and out-planting was conducted under authority of federal permits TE014497-16 from the U.S. Fish and Wildlife Service and HALE-2007-SCI-0012 from the U.S. National Park Service and state permits P-243 and MDF-090216SUP from the Hawaiʻi Department of Land and Natural Resources.

### Analyses

#### Out-plant growth and survival

The influence of seed source elevation on growth of plants in the greenhouse was evaluated with a linear mixed model. Rosette diameter immediately prior to out-planting was the response, source elevation (mid, high) was a fixed effect, and maternal plant nested in source elevation was included as a random effect. Potential differences in mean rosette diameter among plants allocated to each out-plant site was tested with a one-way ANOVA.

To evaluate the potential influences of seed source elevation and out-plant site on individual plant survival, we used a generalized linear mixed model with survival at the end of the fifth dry season (57 months post out-planting) as the binary response, source elevation and out-plant site as fixed factors, and maternal plant as a random factor. Similarly, the influence of these factors on out-plant growth was assessed with a linear mixed model, with RGR from the time of out-planting until the end of the fifth dry season (among surviving plants) as the response, and source elevation and out-plant site as fixed factors, and maternal plant included as a random factor. One site (Laie) was excluded from the RGR model because only one plant survived and thus had a single RGR measurement at 57 months post-planting. Several plants flowered towards the end of the study period, after which they died as a result of monocarpy. For the purposes of survival analyses, these plants were treated as living because their deaths did not appear to be stress related. RGR for these plants was calculated using their final pre-flowering diameter measurements.

In evaluating climatic influences on plant performance, our goal was to identify simple and robust relationships that could subsequently be spatially modelled. Because plants assigned to each out-plant site were completely balanced with respect to the seed source elevation, and because this individual-level factor was found to have little effect on survival and growth after out-planting (refer to “Results”), we evaluated plant outcomes as site-level metrics. Thus, plant survival was evaluated as the cumulative percent survival at each site at the end of the study period (57 months post planting), and growth was evaluated as mean RGR at each site from the time of planting to the end of the study period. This approach allowed us to use simpler general linear models that focused on relative climatic suitability of sites over the course of the study, our primary objective, rather than on the specific weather conditions contributing to individual plant mortality occurring over shorter timeframes. It also yielded coefficient of determination (*R*^2^) values, which were useful for comparing amounts of variation explained by competing models. For each response, we evaluated models that included up to two of the five climate variables measured or calculated at each site (AT, RF, RH, SM, and VPD), plus their interactions. Daily climate means (or totals in the case of RF) were averaged over the entire study period. Three combinations of variables were excluded because of high collinearity (*r* > 0.7; RH and RF, RH and VPD, and RF and VPD), resulting in 19 possible models for each response, which were ranked according to lowest AICc score. We also considered a logit transformation of the cumulative survival response to represent the bounded nature of the percent survival metric. However, we found the logit response to be similar but with a slightly poorer fit because values near upper and lower survival bounds are spread out on the transformed scale, giving these values undue influence that was not biologically plausible and did not match patterns in the data. Hence, we used the untransformed survival response to fit models and then bounded subsequent suitability projections (described in spatial modeling below).

For the percent survival response, two models had AICc scores within ~ 4 of the top model (refer to Results), indicating reasonably similar fits among this three-model candidate set^42^. One of these (AT + RF) had similar explanatory power (measured by *R*^2^) as the top model while also readily permitting spatial interpolation over the landscape with existing climate rasters. This model was therefore chosen for subsequent spatial modelling (described below). For RGR, the range in mean responses among sites was less informative (refer to Results), so spatial modelling of RGR was not subsequently conducted.

#### Spatial modelling of empirical results

To model habitat suitability for out-planting across Haleakalā Crater and surrounding areas, we first defined a relative climatic suitability index, ranging from 0 to 1, as equivalent to the cumulative percent survival estimated by the AT + RF model. Actual survival of future out-planting efforts will depend on timing relative to shorter-term weather patterns, specific micro-sites selected, the watering regime employed, and other factors. However, because survival in our experiment varied from just above 0% to almost 100% over a nearly 5-year period and because AT and RF gradients explained nearly 90% of this variability (refer to Results), we argue that the resultant patterns should generate good estimates of relative suitability across the landscape, all other factors being equal.

To produce a map of estimated current relative climatic suitability, we applied the AT + RF model to 250-m gridded rasters of mean AT and total RF for the island of Maui^43^, by plugging in AT and RF values averaged over the same 57 months as the study period for each grid cell. To confirm that the AT and RF rasters accurately represent the study area, we compared mean annual AT and mean annual RF at each out-plant plot climate station with the corresponding raster values for each year of the study period. These aligned well, with only small biases apparent in the raster data (Figs. S2, S3). We then corrected these biases by applying the linear regression relationships between the station data and raster data before applying the survival model to the rasters. We restricted the projection area to habitat deemed appropriate for silversword restoration, which we defined as above approximately 2000 m elevation on the southern and western flanks and above treeline on the northern and eastern flanks of Haleakalā volcano. The climatic suitability index was bounded between 0 and 1 by setting all projected suitability values < 0 and > 1, to 0 and 1, respectively, which represented relatively few grid cells. For this and the following projections, we also examined alternate results generated from a AT + RF model fitted to logit-transformed survival; these were very similar to the percent survival model results and are not discussed further.

To estimate how relative climatic suitability may have changed over the past few decades, we generated maps for each consecutive five-year period from 1995 to 2020 by applying the AT + RF model to rasters averaged over 57 month periods ending in October of each period. The first period possible was 1995 because available rasters begin 5 years earlier in 1990. We then regressed the predicted climatic suitability index over time for each grid cell, and mapped the slopes of these fits to create a picture of the rate of change in suitability across the study area (scaled to an annual rate of change). Because these temporal data exhibited little serial autocorrelation, as judged with autocorrelation function plots, we used simple linear regression. Due to the small sample size for each time series (i.e., only 6 non-overlapping 5-year periods), we evaluated statistically significant trends at alpha values of 0.1 and 0.2 when assessing spatial patterns. We also projected these trends 1–3 decades into the future, producing climatic suitability projections for 2031, 2041 and 2051, by simply extrapolating the observed 30-year trends forward. All analyses were performed in SAS JMP Pro 16.1.0 and the nmle package in R version 4.2.0^44^. All spatial modelling was performed using the terra package in R^45^.

#### Standard SDM using occurrence data

To compare our empirical approach with widely used occurrence-based SDM methods, we made an effort to create a robust SDM for the Haleakalā silversword using best available methods. Presence locations included in the model were based on the centroids of 101 population areas that had live individuals observed in the latest full census of the species conducted in 2013–2014^28^. All grid cells within the target region that were > 250 m from these delineated population areas (n = 2476) were identified as potential absences (i.e., pseudo-absences) to be sampled during model fitting. As with the empirical modelling approach, we fit our models based on AT and RF alone, using 250-m gridded rasters of long-term climate means widely used in Hawaiʻi (i.e., MAT and MAP^32,43^). We used an ensemble modeling approach that combined species-specific tuned boosted regression trees and Maxent models, two widely used SDM approaches shown to give reliable results^46^. A total of 80 models was created, from which we generated a final ensemble model that was a weighted average of all models in which individual model influence was directly related to its training stress score (TSS) evaluation metric^47^. This ensemble modeling approach has been shown to improve the reliability of SDMs^48^. Additional details of SDM methods are provided in Supplementary Information.

## Results

### Out-plant growth and survival

Rosette diameter immediately prior to out-planting was not significantly influenced by seed source elevation (*F* = 3.312, *p* = 0.107), and there was no significant difference in mean rosette diameter among plants allocated to each out-plant site (*F* = 0.876, *p* = 0.537). The overall mean rosette diameter at the time of out-planting was 15.5 ± 0.2 (SE) cm.

**Figure 2:**
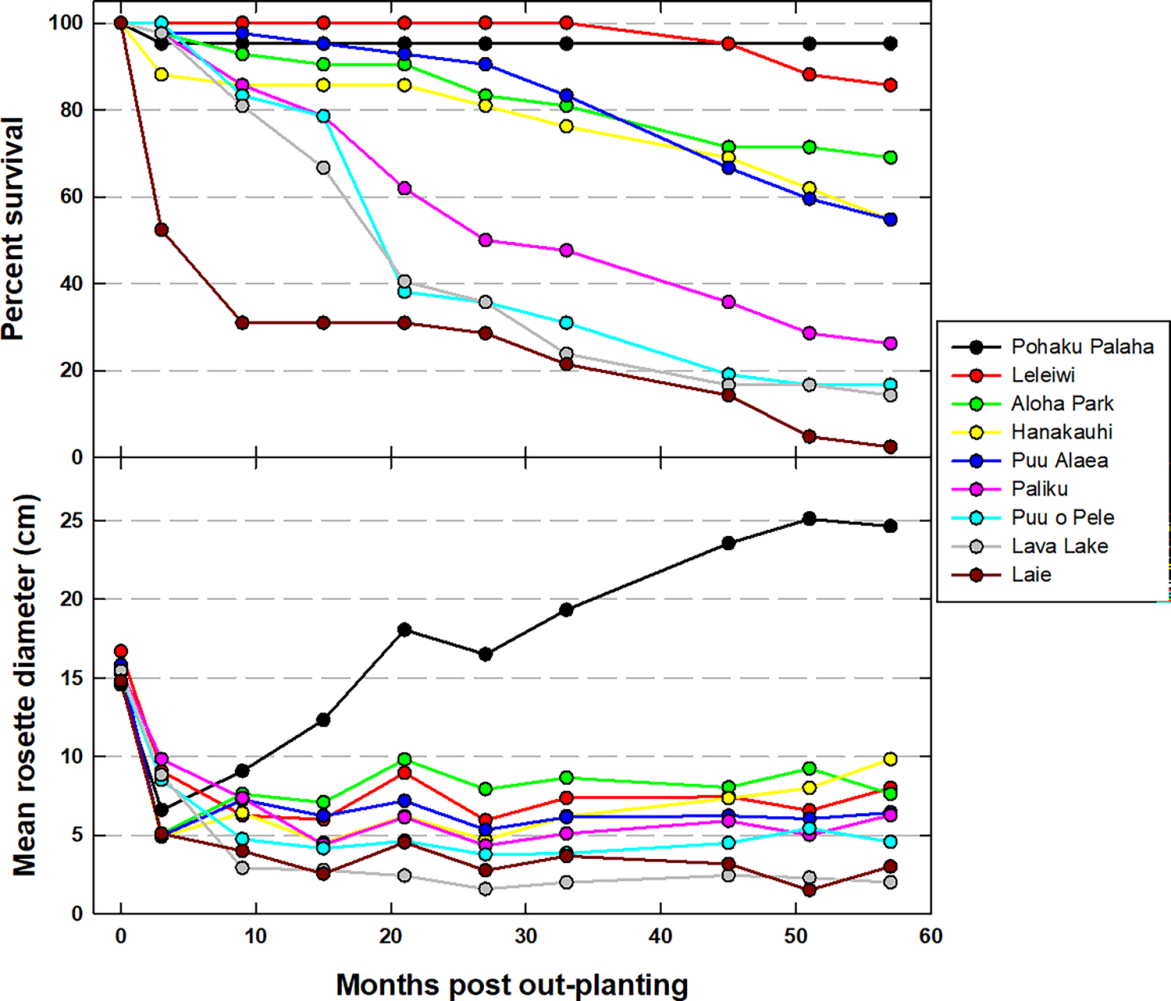
Percent survival (top panel) and mean rosette diameter (bottom panel) of out-plants at each site at the end of each wet and dry season over the course of the study period.

At the end of the study period, 57 months after out-planting, individual plant survival was not significantly associated with the source elevation of seeds (*X*^2^ = 0.63, *p* = 0.429). However, individual plant survival was significantly influenced by out-plant site (*X*^2^ = 175.10, *p* < 0.0001), which was reflected in strong variation in percent survival among sites, which ranged from 2.4 to 95.2% (Fig. 2, top panel).

Plants at all out-plant sites shrank greatly in the initial 3 to 9 months after out-planting, after which mean rosette diameter was relatively stable at all sites except Pohaku Palaha, where plants fully recovered and eventually exceeded their initial mean size (Fig. 2, bottom panel). Individual plant RGR from the time of out-planting to 57 months later was not significantly associated with the source elevation of seeds (*F* = 0.146, *p* = 0.721). As with survival, individual RGR was significantly associated with out-plant site (*F* = 26.388, *p* < 0.0001). This was largely driven by a mean RGR at the Pohaku Palaha site that was significantly higher than all other sites; in addition, mean RGR at the Lava Lake site was significantly lower than all other sites except Puʻu o Pele (Fig. S4).

**Figure 3:**
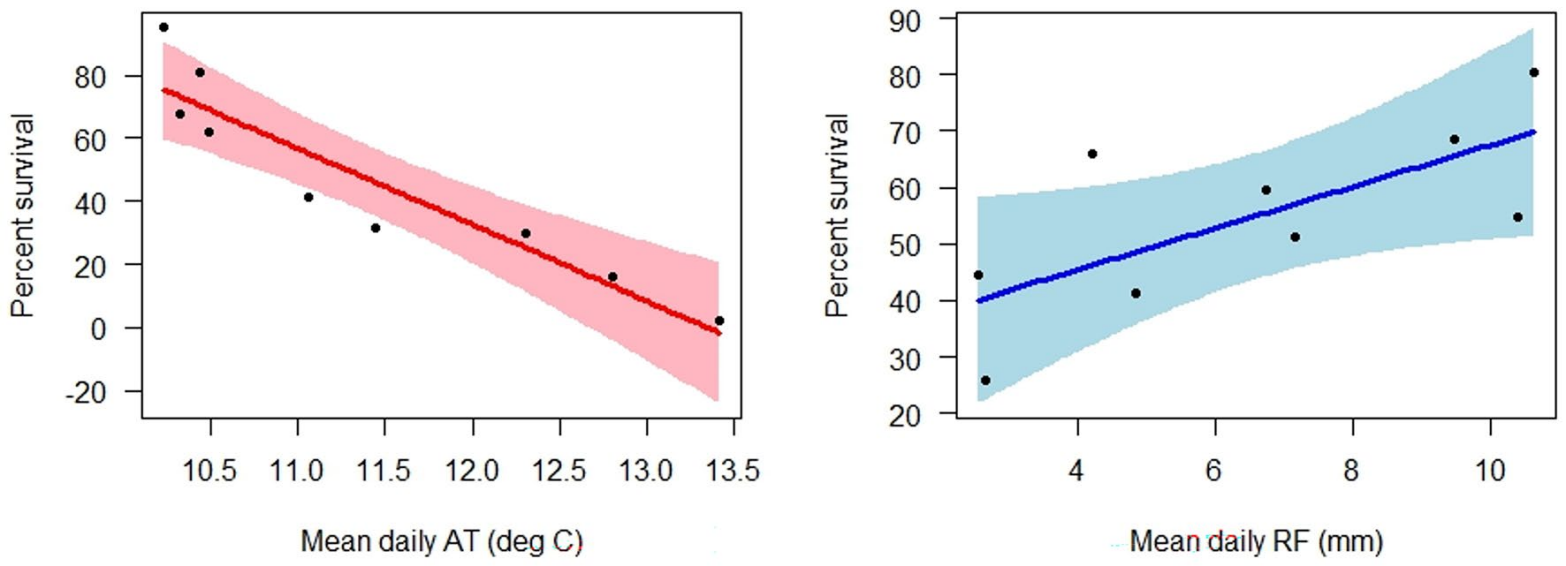
Fitted relationships between percent survival and AT (Left panel, *F* = 37.99, *p* = 0.0008), and percent survival and RF (right panel, *F* = 6.25, *p* = 0.0466) in the AT + RF model at the end of the study period. Each plot shows 95% confidence bands and partial residuals.

Three climate models had reasonably similar fits to percent survival of out-plants at the end of the study period: AT, AT + SM, and AT + RF (Table S1). Of these, AT + SM had the lowest AICc score and the highest *R*^2^ of the three models. However, SM can be spatially idiosyncratic, depending strongly on local edaphic conditions that may not vary in a systematic fashion^49^. This makes accurate spatial mapping of this variable difficult, especially on Haleakalā where numerous distinct lava flows and cinder deposits create a mosaic of substrates of different ages and compositions^50^. In contrast, both AT and RF vary systematically across the top of the mountain and have been spatially interpolated at fine scales in existing climate raster products^43,51^. Although delta AIC cutoffs of 2 or 4 are commonly used to exclude models from candidate sets, the AT + RF model had a delta AICc value only slightly above that (4.24, Table S1), while yielding very high explanatory power that was similar to that of the AT + SM model (*R*^2^ = 0.88 for AT + RF versus *R*^2^ = 0.93 for AT + SM, Table S1; the AT model had lower explanatory power, *R*^2^ = 0.76). In fact, this two-term model (percent survival = 298.48 – 24.22*AT + 3.68*RF) exhibited very good fit to the survival data (Fig. 3). To confirm that seed source did not have an important influence on these climate relationships, we constructed a model in which source elevation and its interactions with AT and RF were fit to percent survival (calculated among plants from each source elevation); AT and RF remained significant (*p* < 0.0001 and *p* = 0.004, respectively), while source elevation (*p* = 0.536) and its interactions (*p* = 0.258 for interaction with AT, *p* = 0.918 for interaction with RF) were not significant. We therefore used the AT + RF model fit to percent survival of all plants for spatial modelling of relative climatic suitability.

Seven climate models had reasonably similar fits to RGR of out-plants over the course of the study period: SM, SM + RF, RF, VPD, VPD + SM, RF + AT, and AT (Table S2). The three models containing two variables generally had higher explanatory power (*R*^2^) than the single-variable models. Nevertheless, there was little useful variation in mean RGR among sites, with all sites except Pohaku Palaha exhibiting negative growth, and with most of the negative rates clustered in a small range (Fig. S4). We therefore concluded that spatial modelling of this response would not be very informative.

### Spatial modelling

**Figure 4:**
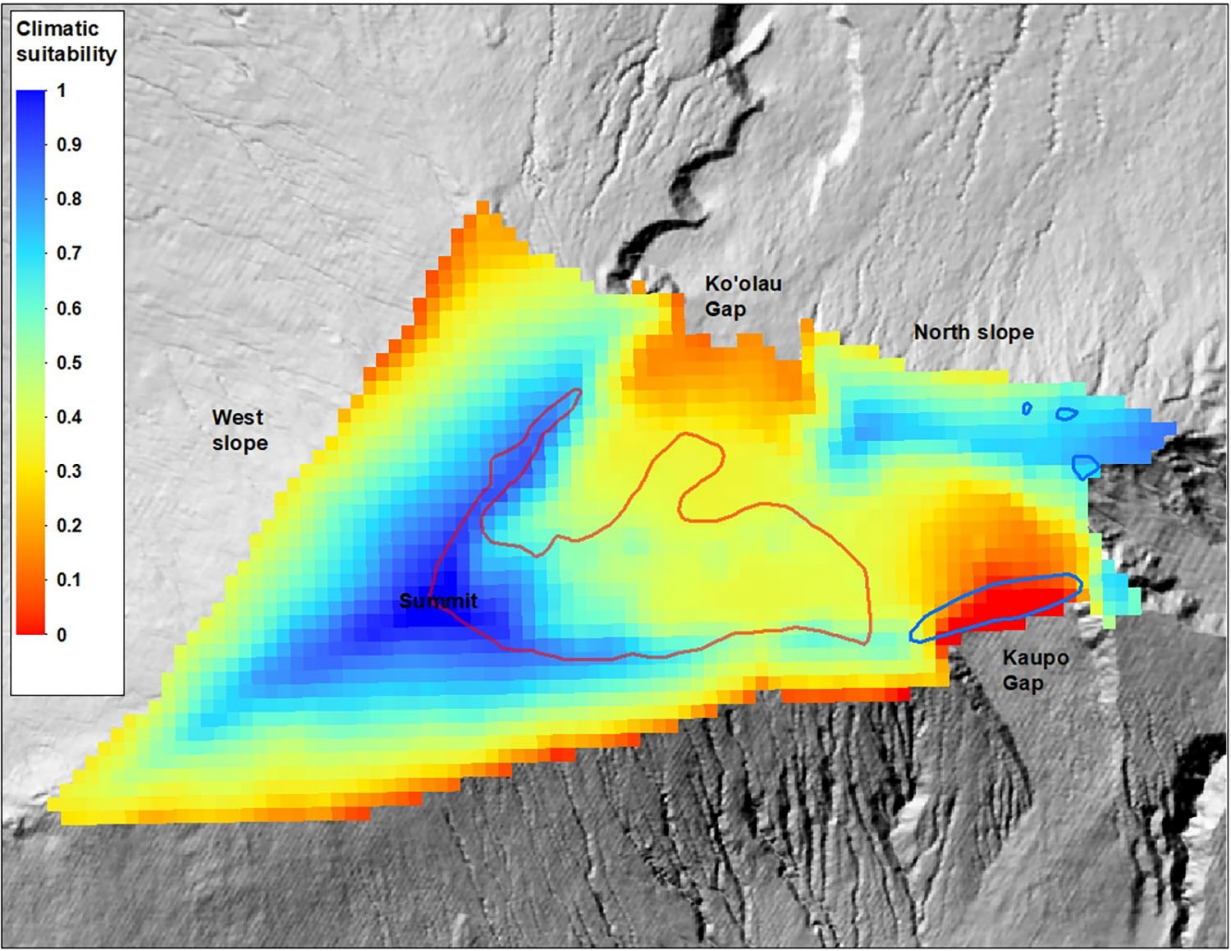
Map of current relative climatic suitability for silversword out-planting across the top of Haleakalā volcano. Red polygon is the current range and blue polygons are estimated historical populations. Map created using ArcGIS software version 10.8.2, https://www.esri.com/en-us/arcgis/products/arcgis-desktop/overview.

Based on the strong link between out-planting survival and climate established by our models above, climatic suitability for silversword out-planting is currently estimated to be high in the summit region of the volcano, with suitability declining with decreasing elevation away from the summit (Fig. 4). The northern crater rim and upper northern slope of the mountain, east of Koʻolau Gap, was also identified as a region of moderate to high climatic suitability. The easternmost portion of this region is covered in thick grassland, however, and the suitability of this habitat type for silverswords is unknown. Several additional smaller regions of moderate climatic suitability on the mountain were identified along the southern crater rim west of Kaupo Gap, and around Kuiki peak east of Kaupo Gap. Notably, only a relatively small portion of the current range is estimated to now have high suitability for out-planting.

**Figure 5:**
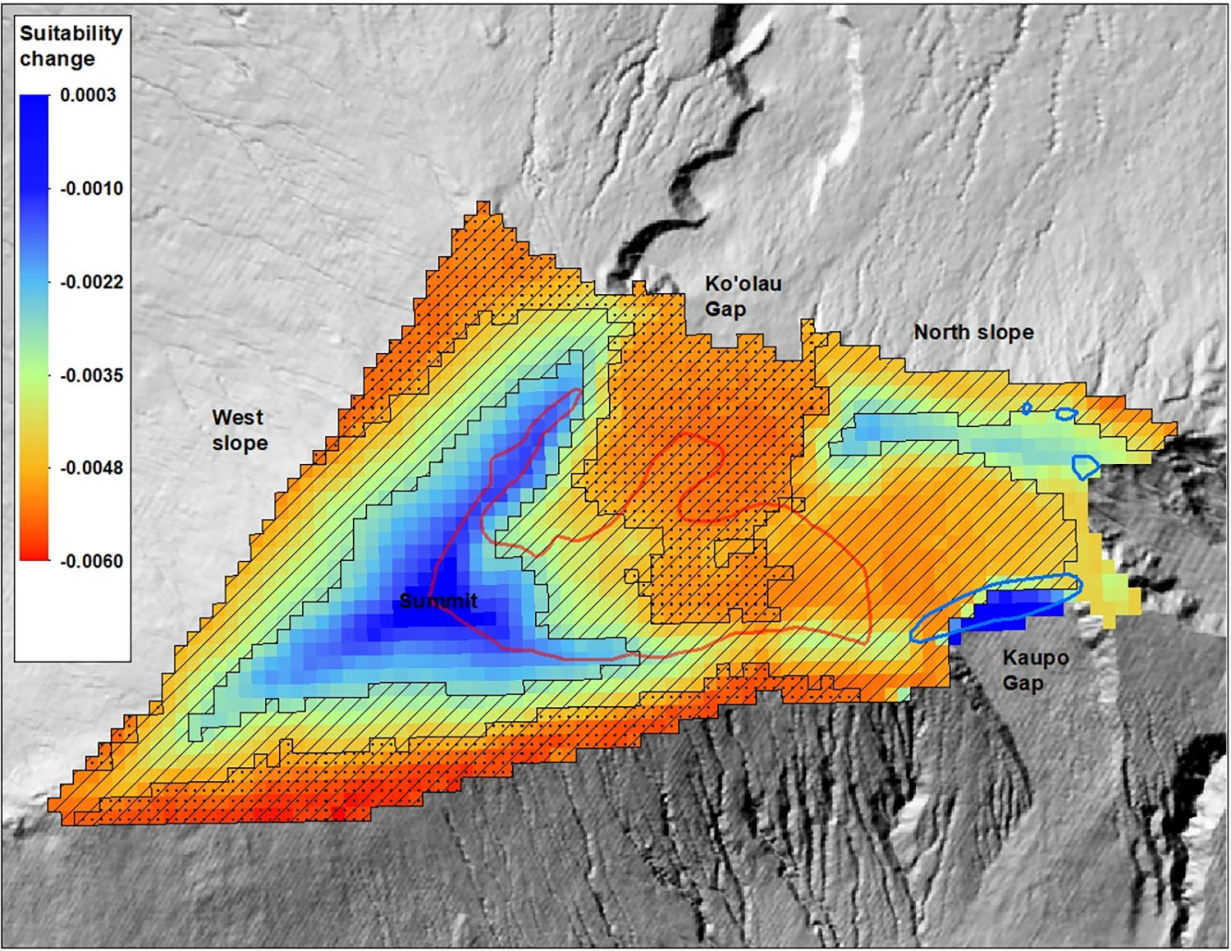
Map of linear trends in climatic suitability from 1995 to 2020, expressed as rate of annual change in the suitability index. Areas with overlaid hatching pattern have trends significant at *α* = 0.2, and areas with overlaid stippled pattern have trends significant at *α* = 0.1. Red polygon is the current range and blue polygons are estimated historical populations. Map created using ArcGIS software version 10.8.2, https://www.esri.com/en-us/arcgis/products/arcgis-desktop/overview.

Based on our six 5-year projections of climate suitability between 1995 and 2020, a very spatially consistent pattern of decreasing climatic suitability is observed across most of the study area (Fig. 5). Rates of decline are largest in lower elevation and drier regions, generally corresponding to areas where current suitability is lowest. Suitability has appeared to be most stable near the summit, where it remains highest. Suitability also has changed little in the lower elevation Kaupo Gap region because this area was consistently unsuitable across the entire time series. Declining suitability trends were statistically significant at alpha levels of 0.1 or 0.2 across most of the lower elevation and drier regions, but trends were not significant in the regions where suitability changed least (Fig. 5).

**Figure 6:**
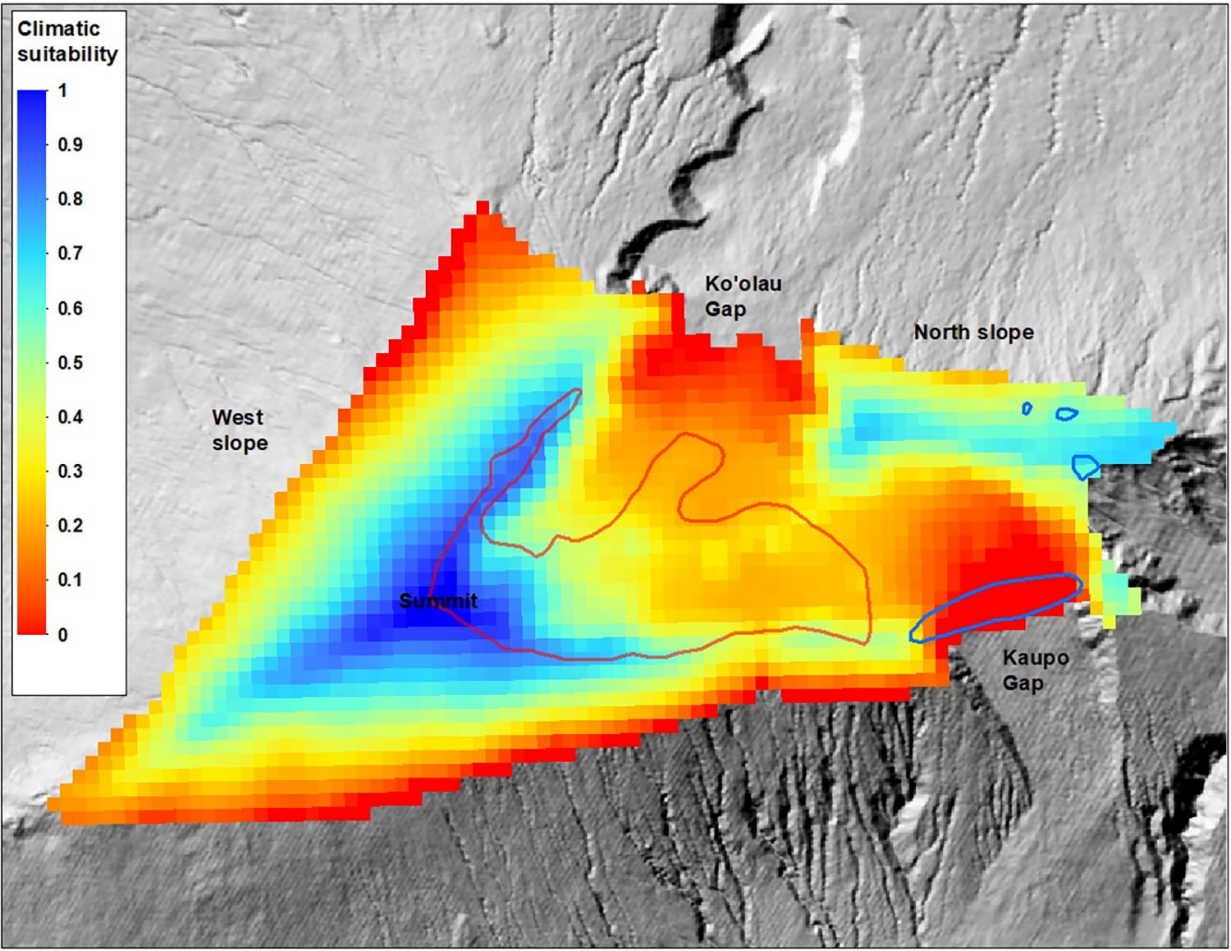
Map of estimated relative climatic suitability for the period ending in 2051. Red polygon is the current range and blue polygons are estimated historical populations. Map created using ArcGIS software version 10.8.2, https://www.esri.com/en-us/arcgis/products/arcgis-desktop/overview.

Future climatic suitability is projected to progressively decline across most of the study area from 2031 (Fig. S5) to 2041 (Fig. S6) to 2051 (Fig. 6). If recent trends continue, by 2051 the summit region will remain the largest area with moderate to high suitability, while the two smaller areas to the east and west of Kaupo Gap are predicted to diminish substantially in suitability. The region on the upper northern slope is predicted to retain a sizeable area of climatically suitable habitat.

### Standard SDM

The average TSS evaluation metric across all 80 computed models was 0.828, indicating a good statistical fit to the presence data, and with similarly high metrics using alternative evaluation methods such as ROC (0.964) and Kappa (0.489). Mean variable importance was 0.707 for MAP and 0.303 for MAT, indicating variability in precipitation across the study area had a stronger relation to the distribution of silverswords across the study area. Nevertheless, the ensemble response curves for the species show a clear and narrow habitat preference for the species, based on the available data (Fig. S7). As expected, the projection of the ensemble model over the study area shows that the predicted presence of the species covers all population areas with existing occurrences, along with some adjacent regions with similar climatic conditions (Fig. S8).

## Discussion

### Current and future suitability for silversword restoration

Climatic suitability for silversword out-plants is predicted to be high in a relatively large portion of the summit region of Haleakalā volcano by our empirical model, including substantial habitat outside the current range. Provided that recent climate trends continue, this region is also projected to retain high suitability over the next few decades, which is important given the relatively long generation time of this species^29^. Much of this summit region has historically been unprotected from feral ungulates, so these results show that the current efforts to fence and restore portions of the area can advance conservation in this region. Our model also identified a second, smaller region on the upper northern slopes of the mountain that currently has relatively high suitability for out-planting, but where plants no longer occur. Although this region is predicted to diminish in suitability somewhat over the coming decades, re-establishment of silverswords on this portion of the mountain could nevertheless provide an important second population to help counteract the recent decline of the species. The northern slope area also includes substantially wetter habitat relative to the summit region, and may therefore offer some added insurance against unexpected and uncertain future changes in precipitation^52^. Because all areas identified as possessing relatively high climatic suitability are either within or adjacent to the known historical distribution of Haleakalā silverswords, out-planting in these regions should pose little risk to other rare and vulnerable species.

Like most standard SDMs, our empirical modelling approach yields estimates of spatial variation in suitability. This can help guide management decisions regarding where to focus restoration or translocation activities, but it does raise the question of what level of relative suitability is needed to ensure a reasonable probability of establishing self-sustaining populations. While higher values are always preferable, without additional information the selection of a threshold suitability value may be largely arbitrary. Fortunately, extensive demographic data exist for the Haleakalā silversword that can provide guidance in our case. Although nursery-grown out-plants can differ from wild plants in ways that may influence their growth and survival, an example being small rooting volumes constrained by pots^53^, these differences are expected to diminish over time, and demographic rates among wild populations can still offer useful insights into expected dynamics following out-planting. This is especially true regarding the offspring of out-plants, which are most relevant to the longer-term success or failure of a restoration or translocation effort. On Haleakalā, regions above roughly 2500 m elevation have supported stable to positive population growth rates of wild silverswords over the past several decades^28,29^. Furthermore, demographic models developed for this species^29^ suggest that below this elevation, proportional survival rates of at least 0.7, among roughly 1-year-old plants over a 5-year period, would typically result in stable populations (assuming historically average vital rates among other stages). Therefore, restoration activities may be more successful if focused above ~ 2500 m elevation, or, when below this elevation, in regions with relative climatic suitability values above a 0.7 threshold.

**Figure 7:**
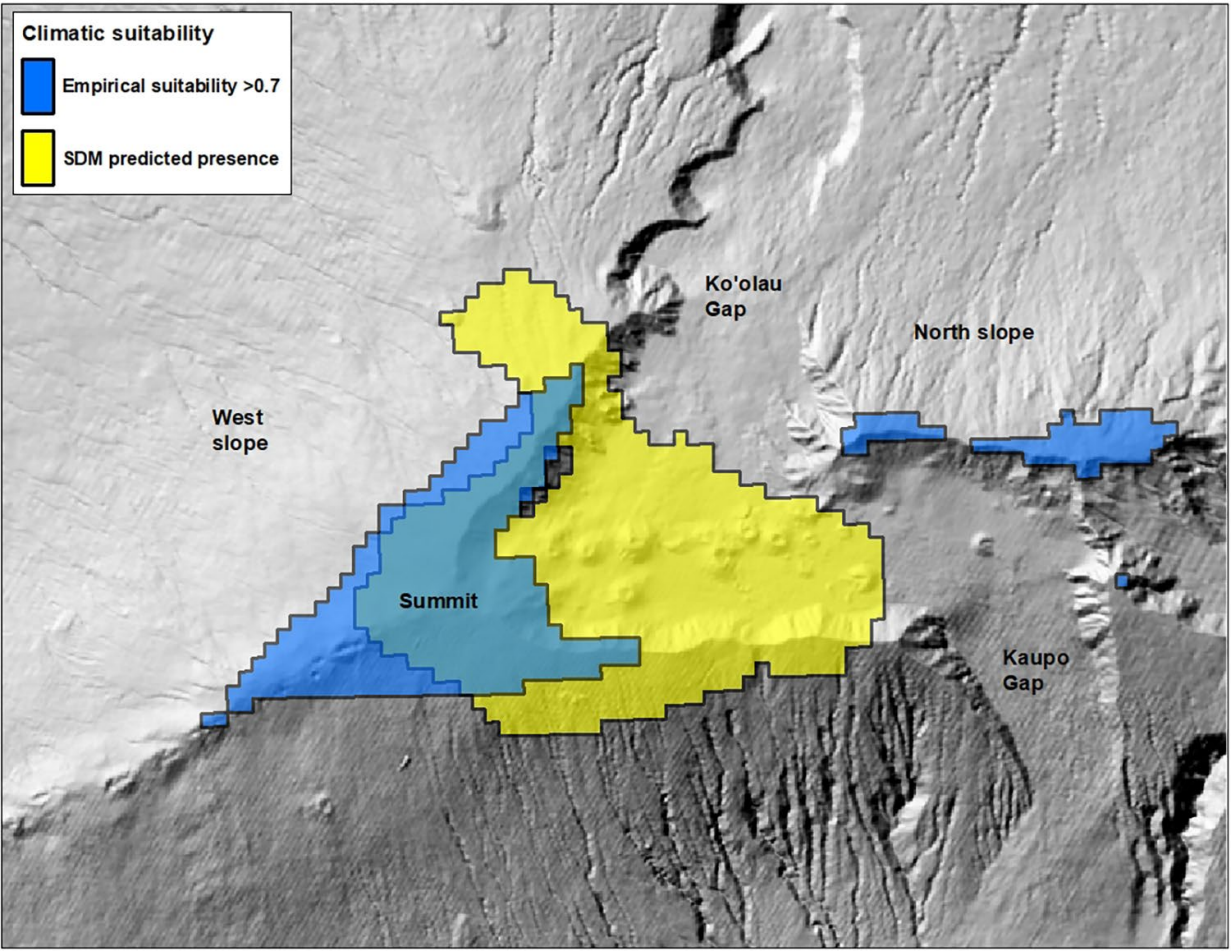
Areas predicted to be most climatically suitable for out-planting by the empirical model (blue polygon, defined as suitability > 0.7, refer to text) compared to areas predicted to be climatically suitable for silversword presence by the SDM (yellow polygon). Areas of overlap are gray-green. Map created using ArcGIS software version 10.8.2, https://www.esri.com/en-us/arcgis/products/arcgis-desktop/overview.

Unfortunately, this threshold predicts that most of the current silversword range (~ 67%) is probably now largely climatically unsuitable for restoration activities. Possible exceptions include favorable micro-sites like seeps, where higher local rates of survival^27^ may indicate that out-planting efforts could help promote persistence over the longer-term. On the positive side, the suggested threshold predicts that climatically suitable habitat greater in size than this area lost exists across several areas outside the current range, at least for the time being. Notably, the areas predicted as most climatically suitable for restoration activities by the empirical model differed strongly from those predicted to be suitable for silversword presence by our standard SDM (Fig. 7). The SDM failed to recognize that climatic conditions across much of the current range are now poor for both out-plants and the wild population^28,29^, and was unable to identify the climatically favorable region on the north slope of the volcano. Although contrasts between standard SDMs and empirical estimates of habitat suitability are seldom explored in past research, similar discrepancies have been observed elsewhere^54^. Our research extends this line of inquiry by linking such discrepancies to habitat shifts driven by climate change, thereby broadening the relevance of these findings in light of the ongoing and anticipated impacts of climate change on a diverse array of species globally^55^.

### Model performance and caveats

Our empirical climatic suitability model had remarkably high skill in spite of its simplicity, with mean daily AT and RF explaining nearly 90% of the variation in out-plant survival across sites. The remaining variation was likely influenced by local plot characteristics, such as edaphic features. For example, post-hoc data exploration found that a model that includes SM in addition to AT and RF explains nearly 98% of the variation in percent survival (Figure S9). Because our sensors measured SM only to a depth of 16 cm, they seem unlikely to accurately represent total soil water availability to plants, especially as they aged and presumably expanded their rooting volumes. Nevertheless, these results suggest that our SM measurements may reflect relevant differences in soil hydrologic properties among sites that are independent of RF, as these two variables were not closely correlated (*r* = 0.251). Although it would be difficult to map such small scale variation in soil characteristics across the landscape, soil properties can be considered when evaluating specific out-planting sites.

Similarly, other biotic and abiotic factors may warrant consideration when using this empirical approach in more complex ecosystems^18^. The Haleakalā silversword example benefits from the simplicity of the system, where strong environmental gradients prevail and few biotic interactions appear to impinge strongly on young plant survival. In addition, seed provenance had little influence on patterns of survival, at least among the source regions tested. This is consistent with prior work, which found that while heritable differences in phenotypic traits related to drought resistance exist across the silversword range, this variation does not often lead to strong differences in survival of out-plants^31^. In cases with more pronounced local adaption, however, incorporation of population variation into suitability models could produce suitability maps specific to each population or lineage^17,22,56^.

A potential drawback of our empirical approach is that results would likely vary with each experimental iteration, owing to inconsistent environmental conditions, calling into question the universality of the suitability predictions. However, we hypothesize that as long as the study period represents a substantial fraction of the organism’s life cycle or most vulnerable stage and includes periods climatically stressful enough to induce strong variation in performance across sites, the results are likely to be fairly robust. Furthermore, provided the performance metric is rescaled to span the full range (or close to it) of a relative suitability index (i.e., 0 to 1), even experimental results that are divergent on an absolute scale are likely to produce similar relative suitability maps. In our case, the validity of our suitability maps is bolstered by their congruence with patterns observed across the wild silversword population. For example, the upper portions of the current distribution predicted to have highest suitability for out-planting have also exhibited stable to positive annual population growth rates over the past several decades^29^. Similarly, lower portions of the current distribution, which are predicted to have low suitability for out-planting and are estimated to have experienced the largest declines in suitability since 1995, have consistently experienced negative annual population growth rates over the past few decades^29^ and are estimated to have suffered large cumulative population declines that began around 1990^28^.

## Conclusion

A variety of approaches will likely be needed to guide strategies for the many species globally that have been and will increasingly need to undergo range shifts^6^. Standard SDMs remain the most practical approach for modelling predicted responses for many species and even entire communities^57–59^, where data limitations constrain options. The empirical approach presented here requires considerably more effort than modelling methods that rely exclusively on occurrence data, but it illustrates some of the limitations and inaccuracies that can be inherent in the latter. For example, all of the current silversword range is identified as suitable under a standard SDM that includes all contemporary occurrences, a clear inaccuracy stemming from the failure to recognize declining population trends across much of the current range. More broadly, it is still the prevailing practice in standard SDM studies to assume equilibrium between a species’ occurrences and the environmental factors that shape its distribution, save for the recognized challenges of modelling invasive species that are still undergoing range expansion^60^. In the current era of rapid climate change, we may need to increasingly consider whether this equilibrium assumption is violated for native organisms as well, particularly long-lived species whose distributional responses to climate stress will often be delayed.

More complex modelling approaches that incorporate local adaptation, plasticity, and demographic processes can greatly improve SDM performance^18,19,61^, if data exist to parameterize such models. Yet even these may fail to identify suitable habitats that lack climatic analogues within the current range. Accurately recognizing such areas may only be possible by experimentally testing performance beyond the current range, or by developing detailed biophysical models for species^26,62^. In our study, we found that ample moisture can mitigate high temperatures, allowing silverswords on the north slope of the volcano to thrive at lower elevations if rainfall exceeds values within the current range. In addition to the foregoing advantages, our empirical approach is particularly effective in situations where restoration and possibly translocation can ensure species persistance in a changing climate. This is because unlike strict modelling exercises, it explicitly evaluates dynamics critical to the restoration process. In the case of plants, similar insights might also be gained, with potentially lower effort, by conducting analogous seed-sowing experiments instead of out-planting experiments^24^, or by incorporating careful monitoring into ongoing out-planting management actions.

### Supplementary Information

Supplementary Information.

## Data Availability

Data are available from corresponding author upon request.
