# Supplementary material for: Empirical estimation of habitat suitability for rare plant restoration in an era of ongoing climatic shifts
Source: Sci Rep. 2023 Nov 7;13:19257. doi: 10.1038/s41598-023-46793-7 (PMC10630363; doi:10.1038/s41598-023-46793-7)
Supplement: Supplementary file 1 — Supplementary Information. [file 41598_2023_46793_MOESM1_ESM.docx]

**Supplementary Information for:**

Empirical estimation of habitat suitability for rare plant restoration in an era of ongoing climatic shifts

Paul D. Krushelnycky^1^, Lucas Berio Fortini^2^, Jeffrey Mallinson^3^, Jesse M. Felts^3^

Affiliations: ^1^Department of Plant and Environmental Protection Sciences, University of Hawaiʻi at Mānoa, Honolulu, HI, USA; ^2^U.S. Geological Survey, Pacific Island Ecosystems Research Center, Honolulu, HI, USA; ^3^Resources Management Division, Haleakalā National Park, Makawao, HI, USA

*Scientific Reports*

Any use of trade, firm, or product names is for descriptive purposes only and does not imply endorsement by the U.S. Government.

**Site selection methods**

The aim of the outplanting experiment was to evaluate plant survival and performance across wide gradients in rainfall and temperature, including in former areas of the Haleakalā silversword (*Argyroxiphium sandwicense* subsp. *macrocephalum*) range that are wetter than the current range. We scouted over 20 potential sites that came close to maximizing the ranges of these two variables across the top of the mountain in areas above tree line and outside thick subalpine grasslands. All potential sites also had very low ground cover to avoid confounding dynamics involving plant competition or facilitation, and substrate in most cases consisted of cinder and small sized clasts that appear relatively young even when surrounding substrate is older. From these potential sites, we selected nine study sites that covered most of the ranges in rainfall (RF) and air temperature (AT) while minimizing the correlation between these two variables:

Figure S1. Relationship between mean annual air temperature (AT) and mean annual rainfall (RF) across the nine chosen study sites. Pearson correlation coefficient = 0.13.

Table S1. Candidate set of models relating climate variables to percent survival of out-plants at the end of the study period, 57 months after planting. AT=air temperature; SM=soil moisture; RF=rainfall.

| model | AICc | Δ AICc | *R*^2^ |
| --- | --- | --- | --- |
| AT+SM | 82.019 | 0 | 0.927 |
| AT | 85.485 | 3.466 | 0.761 |
| AT+RF | 86.263 | 4.244 | 0.883 |

Table S2. Candidate set of models relating climate variables to relative growth rate (RGR) of out-plants from the time of planting to the end of the study period, 57 months after planting. SM=soil moisture; RF=rainfall; VPD=vapor pressure deficit; AT=air temperature.

| model | AICc | Δ AICc | *R*^2^ |
| --- | --- | --- | --- |
| SM | -101.170 | 0 | 0.559 |
| SM+RF | -99.123 | 2.047 | 0.751 |
| RF | -98.862 | 2.308 | 0.430 |
| VPD | -98.348 | 2.822 | 0.396 |
| VPD+SM | -97.850 | 3.320 | 0.713 |
| RF+AT | -97.323 | 3.847 | 0.696 |
| AT | -96.844 | 4.326 | 0.286 |


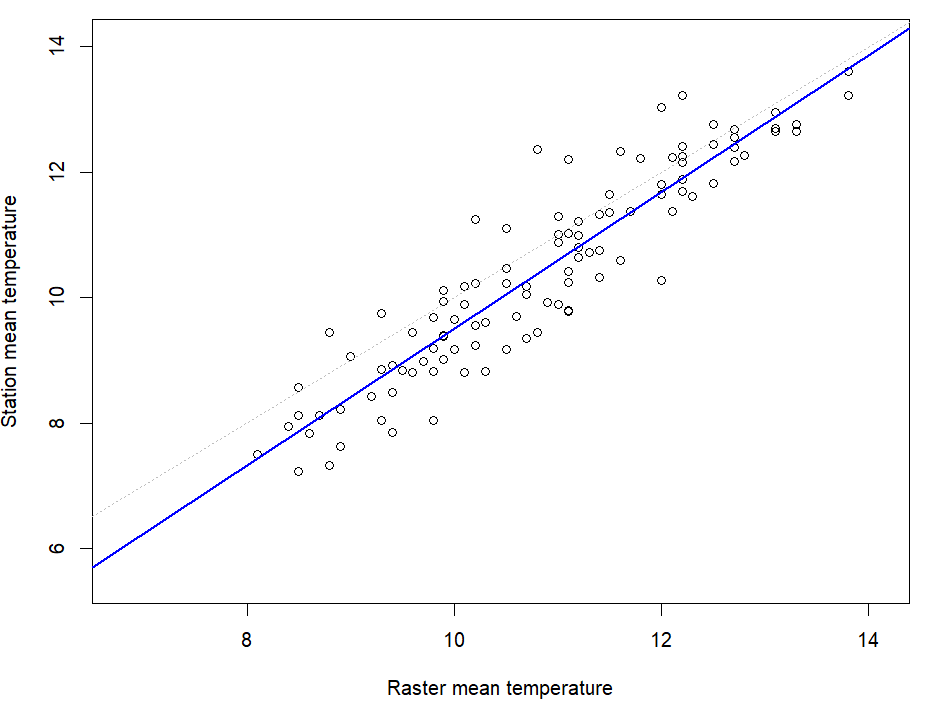


Figure S2. Comparison of mean annual air temperature (MAT) between out-plant plot climate station data and raster data at the corresponding plot locations. Shown are points for all nine plots and for each year during the study period. The blue line is the simple linear regression fit (station MAT = raster MAT*1.092 - 1.411), and the dotted line is the 1:1 relationship.


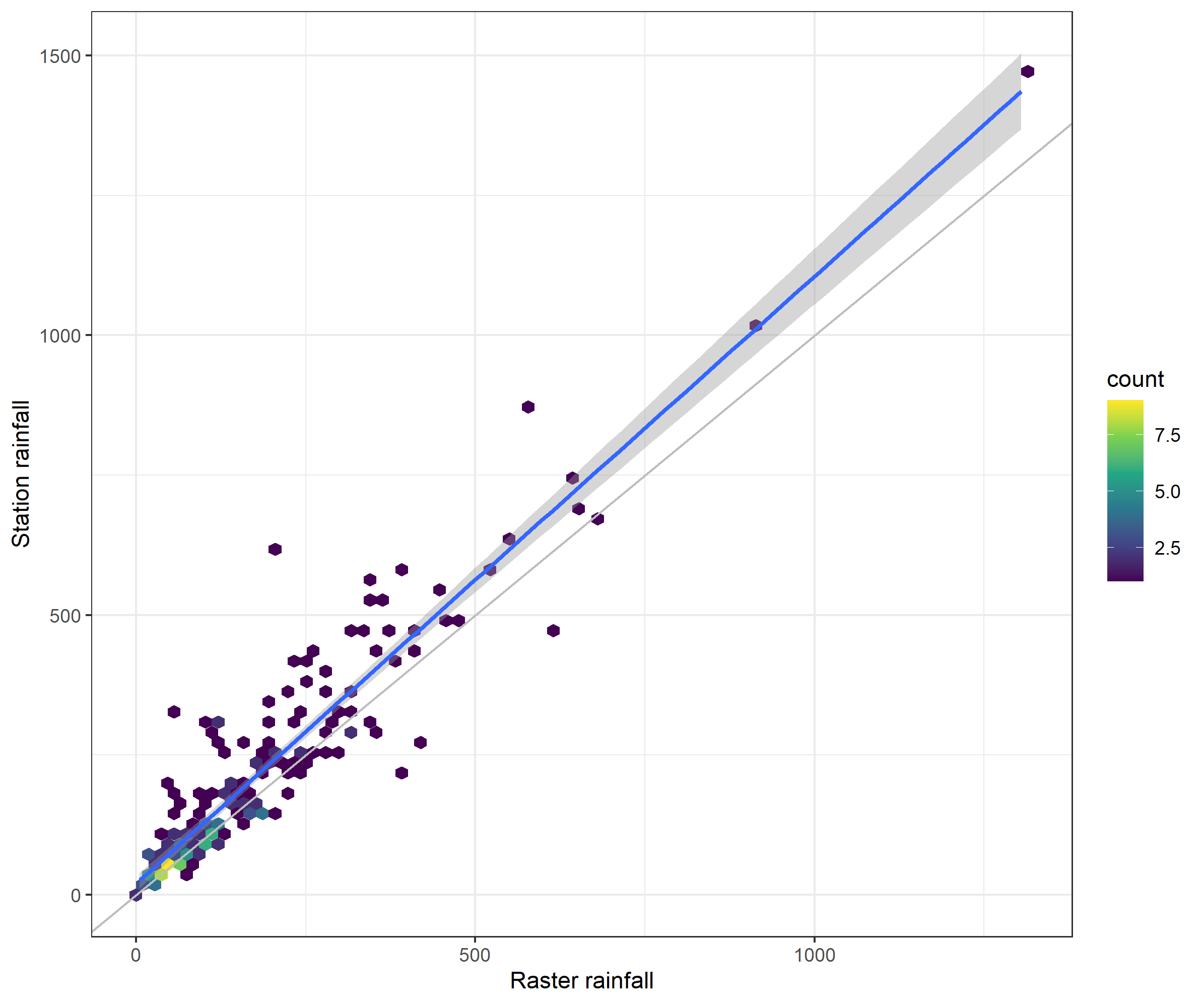


Figure S3. Comparison of mean annual precipitation (MAP) between out-plant plot climate station data and raster data at the corresponding plot locations. Shown are points for all nine plots and for each year during the study period. The blue line is the simple linear regression fit (station MAP = raster MAP*1.085 - 20.453), and the dotted line is the 1:1 relationship.


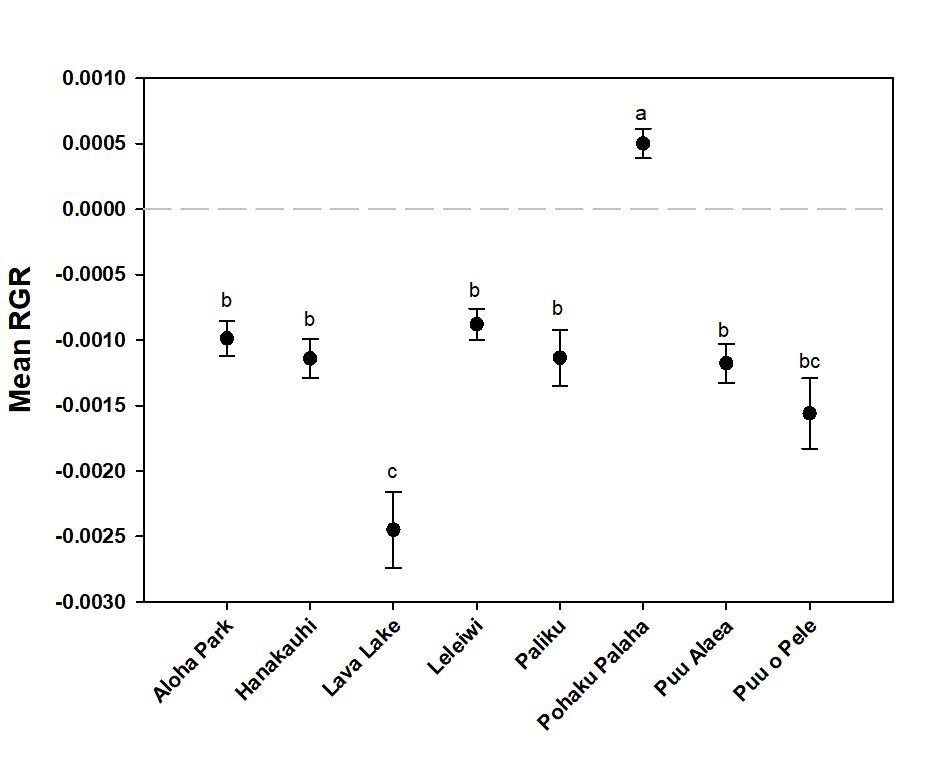


Figure S4. Least square means (±SE) of relative growth rate (RGR) at each site from the time of out-planting to 57 months post-planting. Laie site excluded because only one plant was alive at 57 months.


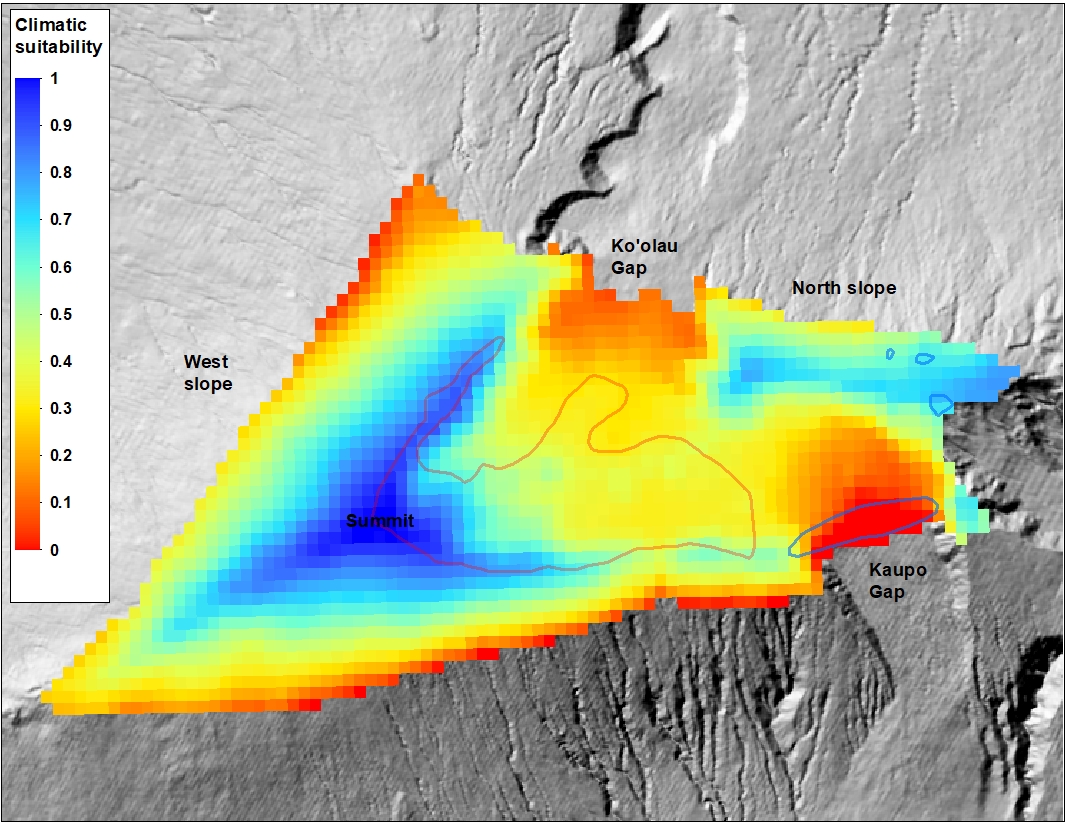


Figure S5. Map of estimated relative climatic suitability for the period ending in 2031. Red polygon is the current range and blue polygons are estimated historical populations.


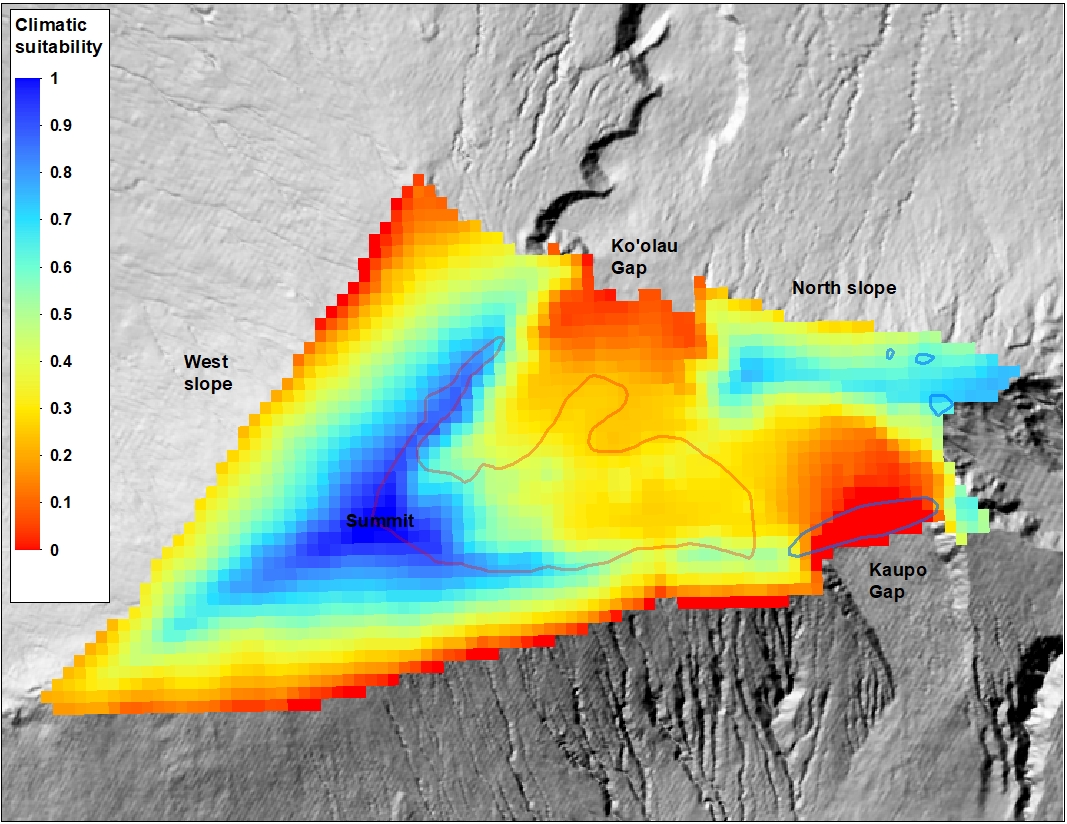


Figure S6. Map of estimated relative climatic suitability for the period ending in 2041. Red polygon is the current range and blue polygons are estimated historical populations.

**Species distribution model methods for Haleakalā silverswords.**

To compare our empirical approach with widely used species distribution model (SDM) methods and results, we made an earnest approach to create a robust SDM for the Haleakalā silversword using best available methods.

**Methods**

For the purposes of comparison, we created a species distribution model (SDM) that represented, as best as possible, the current distribution of silverswords across Haleakalā Crater. Presence locations included in the model were based on the centroid of 101 population areas that had live individuals observed at the latest full census of the species done in 2013. All areas >250 m away from these delineated population areas were identified as potential absences (i.e., pseudo-absences) in our models. Given the 250-m resolution of the analysis, this resulted in a set of 2476 candidate pseudo-absence points to be sampled during model fitting. In all model fitting iterations below, we sampled an equal number of pseudo-absence points to total presence points (i.e., 101). Additionally, to account for the fact that the 101 population areas have a large variation in area and number of individuals, we weighted all presence points in our models by the log of the density of individual plants per ha for each population (i.e., presence point weight = log(population density +1) +1). This ensured all presence points had a weight greater or equal to 1, while all pseudo-absence points had a default weight of 1.

Similar to the empirical model, we limited our model fitting and projection to areas on East Maui above 1800 m in elevation, which encompass Haleakalā Crater and surrounding areas at and above the trade wind inversion height. As in the empirical model, we fit our models based on air temperature and rainfall alone, two climatic predictors known to vary widely across the area and known to be associated with variability in silversword demography (Krushelnycky et al. 2020, Berio Fortini et al. 2021). For the sake of simplicity, we considered only mean annual temperature and precipitation variables (MAT and MAP), as seasonal means correlate strongly with annual means. These were obtained from long-term climatic average 250-m resolution rasters widely used in Hawaii (https://www.hawaii.edu/climate-data-portal/).

We used an ensemble modeling approach that combined species-specific tuned boosted regression trees and Maxent models, two widely used SDM approaches in the literature and shown to give reliable results (Heikkinen et al. 2012). To fine-tune our GBM models, we used the function gbm.step in the R package Dismo (R Core Development Team 2022) to determine the optimal number of trees used to fit models (2000 trees). To fine-tune our Maxent models, we used the function ENMevaluate from the R package ENMeval (R Core Development Team 2022) to determine which data transformations to consider and how much regularization to apply during Maxent model fitting. This resulted in Maxent models with a full set of transforms (linear, quadratic, hinge, product, and threshold) and a regularization value of 2. All other model-fitting parameters were left at Biomod2 defaults.

To avoid an effect from pseudo-absence point selection, we ran 10 rounds of models with different pseudo absence samples. Then for each of these rounds we used a 4 k-fold cross validation where the presence and pseudo absence points were split into 75% for training the model and 25% for evaluating model performance. We used training stress score (TSS) as our evaluation metric as it has been shown to be a more reliable evaluation metric than other commonly used approaches (Allouche et al. 2006). Given the 2 model types, 10 rounds of pseudo absence samples, and 4 k-fold model cross validation runs, this resulted in a total of 80 models created for the species. We then generated a final ensemble model that was a weighted average of all generated models in which individual model influence was directly related to its TSS evaluation metric (Thuiller et al., 2014). This ensemble modeling approach has been shown to improve the reliability of SDMs (Breiner et al. 2015).


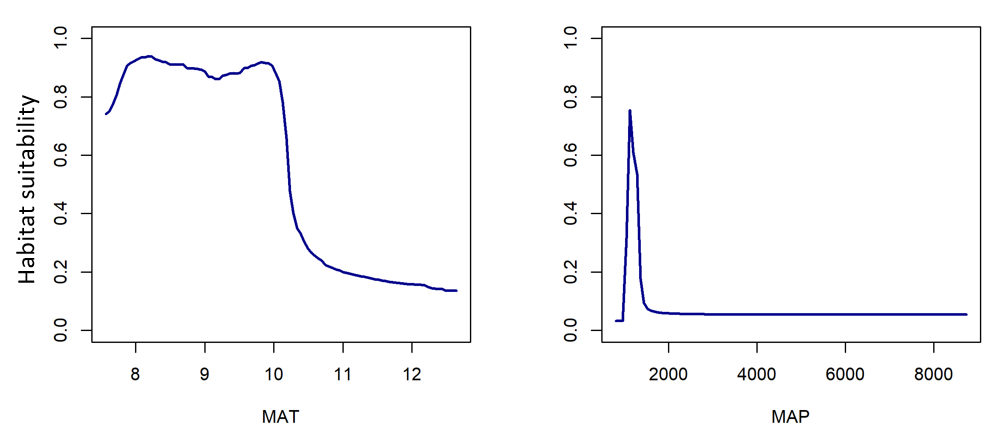


Fig. S7. Haleakalā silversword ensemble response curve indicating how modeled habitat suitability values (on a 0-1 unitless scale) vary with respect to individual environmental predictors.

**
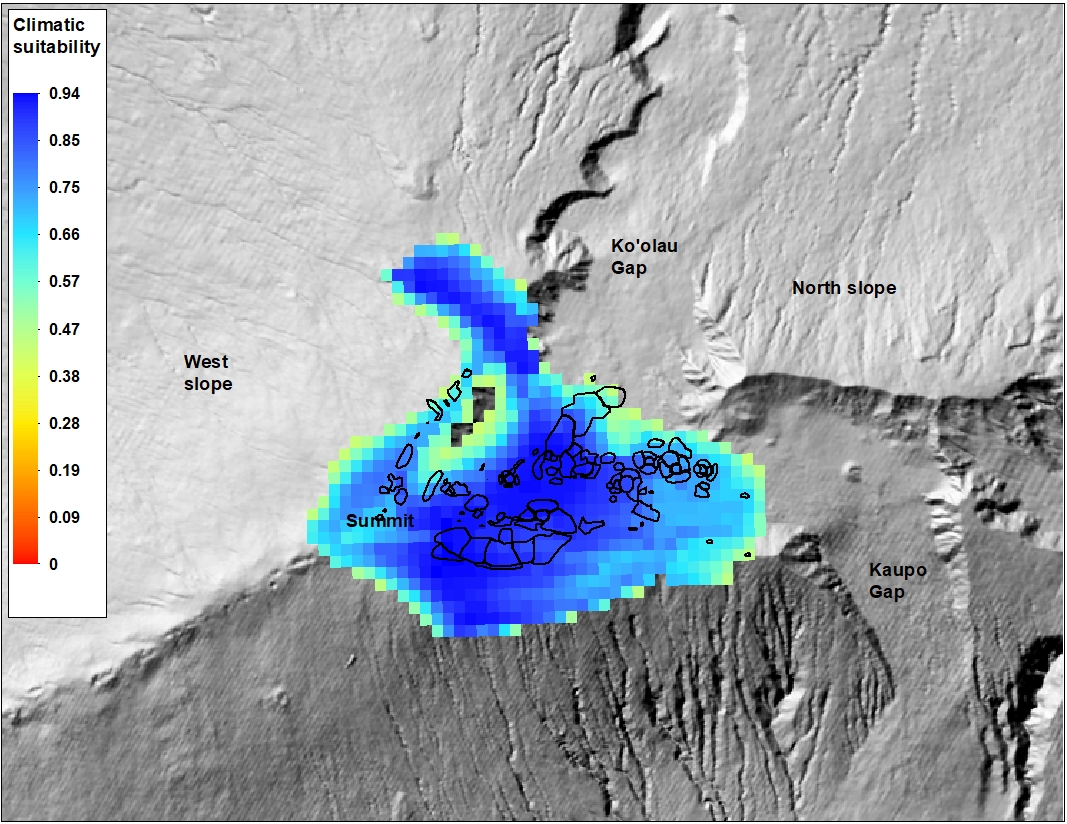
**

Fig. S8. Haleakalā silversword ensemble distribution projection across the study area. Habitat suitability projections are limited to areas the model predicts as presence for the species, from low values of 0.429 to high values of 0.943. Overlay polygons indicate existing population areas used to fit the SDM.


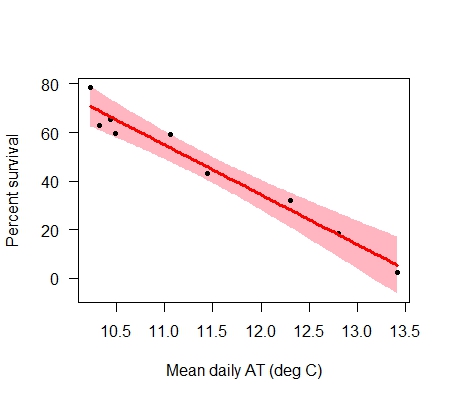

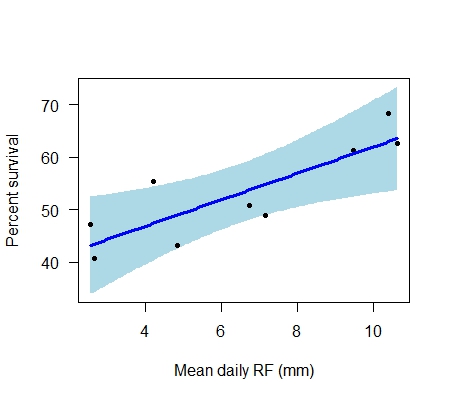


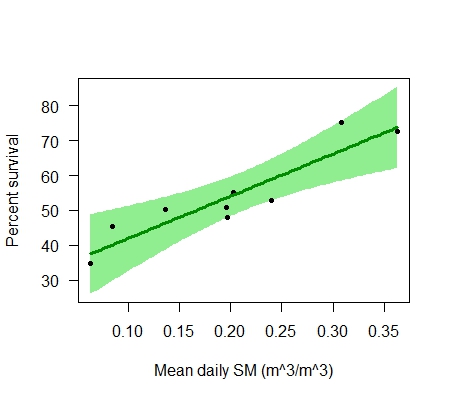


Figure S9. Fitted relationships between percent survival and air temperature (AT) (Top left panel, F = 101.89, p = 0.0002), percent survival and rainfall (RF) (Top right panel, F = 11.47, p = 0.0195), and percent survival and soil moisture (SM) (Bottom left panel, F = 21.39, p = 0.0057) in a AT+RF+SM model at 57 months post-planting (percent survival = 240.06 – 20.55*AT + 2.52*RF + 121.16*SM; r^2^ = 0.978). Each plot shows 95% confidence bands and partial residuals.

**Literature Cited**

Allouche, Omri, Asaf Tsoar, and Ronen Kadmon. “Assessing the Accuracy of Species Distribution Models: Prevalence, Kappa and the True Skill Statistic (TSS).” *Journal of Applied Ecology* 43, no. 6 (2006): 1223–32.

Berio Fortini, Lucas, Paul D. Krushelnycky, Donald R. Drake, Forest Starr, Kim Starr, and Charles G. Chimera. “Complex Demographic Responses to Contrasting Climate Drivers Lead to Divergent Population Trends across the Range of a Threatened Alpine Plant.” *Global Ecology and Conservation* 33 (January 2022): e01954. <https://doi.org/10.1016/j.gecco.2021.e01954>.

Breiner, Frank T., Antoine Guisan, Ariel Bergamini, and Michael P. Nobis. “Overcoming Limitations of Modelling Rare Species by Using Ensembles of Small Models.” *Methods in Ecology and Evolution* 6, no. 10 (2015): 1210–18. <https://doi.org/10.1111/2041-210X.12403>.

Heikkinen, Risto K., Mathieu Marmion, and Miska Luoto. “Does the Interpolation Accuracy of Species Distribution Models Come at the Expense of Transferability?” *Ecography* 35, no. 3 (2012): 276–88. <https://doi.org/10.1111/j.1600-0587.2011.06999.x>.

Krushelnycky, Paul D., Jesse M. Felts, Robert H. Robichaux, Kasey E. Barton, Creighton M. Litton, and Matthew D. Brown. “Clinal Variation in Drought Resistance Shapes Past Population Declines and Future Management of a Threatened Plant.” *Ecological Monographs* 90, no. 1 (2020): e01398. <https://doi.org/10.1002/ecm.1398>.

R Core Development Team. 2022. R: a language and environment for statistical computing, version 4.2.0. https://www.r-project.org/

Thuiller, Wilfried, Damien Georges, and Robin Engler. “Biomod2: Ensemble Platform for Species Distribution Modeling,” July 7, 2014. <http://cran.r-project.org/web/packages/biomod2/index.html>.
